# Identification of microRNAs involved in lipid biosynthesis and seed size in developing sea buckthorn seeds using high-throughput sequencing

**DOI:** 10.1038/s41598-018-22464-w

**Published:** 2018-03-05

**Authors:** Jian Ding, Chengjiang Ruan, Ying Guan, Priti Krishna

**Affiliations:** 1 Key Laboratory of Biotechnology and Bioresources Utilization, Ministry of Education, Institute of Plant Resources, Dalian Minzu University, Dalian, 116600 China; 2 Institute of Berries, Heilongjiang Academy of Agricultural Sciences, Suiling, 152200 China; 3 School of Science and Health, Western Sydney University, Penrith, NSW 2751 Australia

## Abstract

Sea buckthorn is a plant of medicinal and nutritional importance owing in part to the high levels of essential fatty acids, linoleic (up to 42%) and α-linolenic (up to 39%) acids in the seed oil. Sea buckthorn can produce seeds either via the sexual pathway or by apomixis. The seed development and maturation programs are critically dependent on miRNAs. To understand miRNA-mediated regulation of sea buckthorn seed development, eight small RNA libraries were constructed for deep sequencing from developing seeds of a low oil content line ‘SJ1’ and a high oil content line ‘XE3’. High-throughput sequencing identified 137 known miRNA from 27 families and 264 novel miRNAs. The potential targets of the identified miRNAs were predicted based on sequence homology. Nineteen (four known and 15 novel) and 22 (six known and 16 novel) miRNAs were found to be involved in lipid biosynthesis and seed size, respectively. An integrated analysis of mRNA and miRNA transcriptome and qRT-PCR identified some key miRNAs and their targets (miR164d-ARF2, miR168b-Δ9D, novelmiRNA-108-ACC, novelmiRNA-23-GPD1, novelmiRNA-58-DGAT1, and novelmiRNA-191-DGAT2) potentially involved in seed size and lipid biosynthesis of sea buckthorn seed. These results indicate the potential importance of miRNAs in regulating lipid biosynthesis and seed size in sea buckthorn.

## Introduction

Sea buckthorn (*Hippophae* L.) is one of the nutritionally and ecologically most important woody oil plants. Traditionally used for hundreds of years in China and Russia for health-related purposes, sea buckthorn has now gained popularity worldwide due to the unique composition of seed and pulp oil and the abundance of bioactive compounds in fruits, seeds, leaves and bark^1^. The red or orange berries of sea buckthorn contain fleshy pulp rich in vitamin C, carotenoids, flavonoids and monounsaturated palmitoleic acid (up to 39%), and a single seed with an oil content of 8–20%^1,2^, comprising high levels of linoleic acid (30–42%) and α-linolenic acid (20–39%)^3^, carotenoids, flavonol glycosides^4^, tocopherol and phytosterols^5,6^. The bioactive compounds in sea buckthorn products have clinically proven medicinal effects against tumor progression, inflammation, hypertension and gastric ulcers, and can promote wound healing and tissue regeneration^7–11^. The high levels of polyunsaturated fatty acids and the almost 1:1 ratio of linoleic acid to α-linolenic acid, which is considered to be beneficial for human health, makes sea buckthorn seed oil a niche product and warrants the study of seed development and oil biosynthesis pathways. Sea buckthorn sequences associated with fatty acid and triacylglycerol biosynthetic pathways were identified in the mature seed transcriptome and a comparison of gene expression and oil accumulation in seeds derived from four different developmental stages of fruit indicated that oil deposition begins very early in fruit development^2^. Recently, sea buckthorn was reported to produce seeds either sexually or occasionally by apomixis, with the latter ensuring reproduction in the absence of pollination^12^. Aside from these reports there is no other information available on sea buckthorn lipid biosynthesis and seed development pathways.

The seed development and maturation program is, to a major extent, regulated by microRNAs (miRNAs) and their targets encoding functional genes and transcription factors. miRNAs are endogenously encoded small RNAs that post-transcriptionally regulate gene expression. In plants, miRNAs play an essential role in numerous developmental and physiological processes, such as fatty acid biosynthesis^13^, growth and development^14,15^ and responses to various stresses^16,17^, and many miRNAs are conserved across species. The involvement of miRNAs in post-transcriptional regulation of seed or fruit development has been documented in apricot^14^, rice^16^, soybean^18^, peony^19^, *Brassica napus*^13,20^, and *Cichorium intybus*^21^. Examples of functional genes that are related to lipid biosynthesis and targeted by miRNAs, include 3-ketoacyl-ACP synthase (*KAS*) targeted by miR159^13^, 3-ketoacyl-ACP reductase (*KAR*) targeted by miR156 and miR6029^13^, and stearoyl-acyl carrier protein Δ^9^-desaturase6 (*SAD6*) targeted by miR319^22^. Transcription factors play crucial roles in regulating lipid biosynthesis (WRINKLED1^23^, LEAFY COTYLEDON^24^, and helix loop helix (bHLH)^25^) and seed size (ARF^26^, MYB^27^, and CNR^28^). For example, the *Sesamum indicum* bHLH transcription factor binds to E- or G-box elements in the *FAD2* gene promoter and impacts lipid biosynthesis and accumulation during seed development^25^. The ARFs transcription factors involved in seed development are negatively regulated by miR160^29,30^. Accordingly, *ARF10*, *ARF16*, and *ARF17* transcripts were highly increased in the miR160 *foc* mutant during early embryogenesis. miR167, which targets *ARF6* and *ARF8*, is preferentially expressed during late embryogenesis^26^. A double mutation of miR159 (miR159ab), which enhances *MYB33* and *MYB65* expression leads to the formation of small seeds^31^. Additionally, the role of *MYB56* in controlling seed size has been established in *Arabidopsis*^26^. Thus while miRNA-mediated regulatory networks controlling seed oil accumulation and seed development have been revealed in *Arabidopsis*, rice, and maize^26^, little is known in this area in woody oil crops.

miRNAs have been identified in woody oil plants such as olive, oil palm and peony^19,32,33^, but there are currently no reports on miRNAs present in developing seeds of sea buckthorn. To understand oil biosynthesis regulation in sea buckthorn seed, the present study was undertaken to identify miRNAs and their targets in developing seeds of sea buckthorn. Seeds of a high oil content sea buckthorn line ‘XE3’ and a low oil content line ‘SJ1’ were collected at four stages of fruit development: [green (G), green/yellow (G/Y), yellow/orange (Y/O) and orange/red (O/R) color stages], and used for small RNA (sRNA) sequencing. An integrated analysis of mRNA and miRNA transcriptome combined with qRT-PCR identified some novel and previously known miRNAs and their targets potentially involved in lipid biosynthesis. Expression of a subset of novel miRNAs was higher in the low oil content line ‘SJ1’ as compared to the high oil content line ‘XE3’, while the expression of miR164d with the target gene ARF2, which determines seed size, showed the opposite pattern. These results indicate the potential importance of miRNAs in regulating lipid biosynthesis and seed size of sea buckthorn via post-transcriptional mechanisms.

## Results

### Oil content, fatty acid composition and seed size of developing sea buckthorn seeds

**Figure 1:**
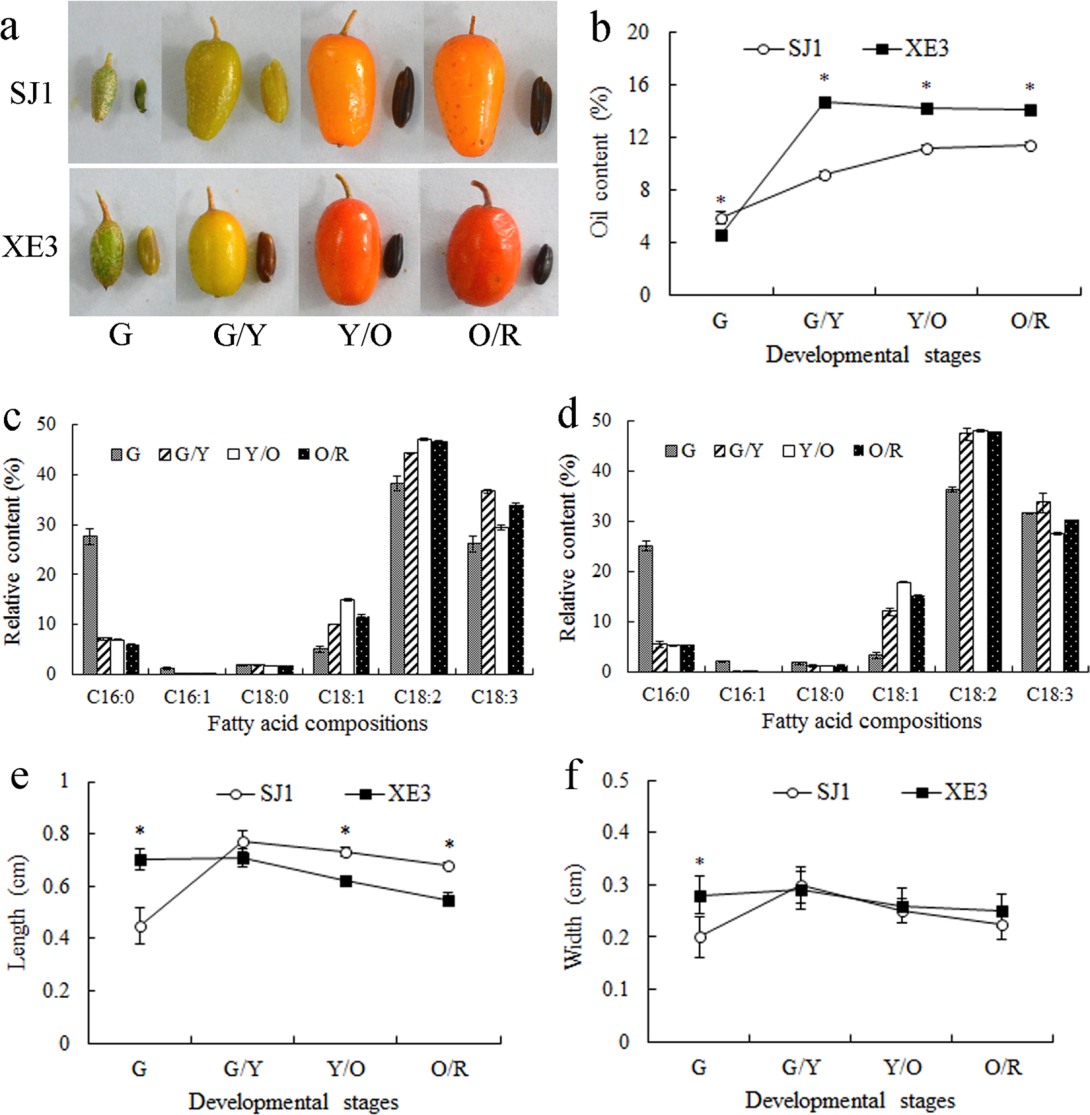
The oil content, fatty acid composition and size of developing sea buckthorn seeds. (**a**) Fruits and seeds of sea buckthorn at four development stages. G, G/Y, Y/O and O/R indicate the developmental stage of the fruit as indicated by color green, green/yellow, yellow/orange and orange/red, respectively. (**b**) Oil contents in seeds from lines ‘SJ1’ and ‘XE3’ at four development stages. (**c**) Fatty acid compositions in seeds from lines ‘SJ1’ at four development stages. C16:0, C16:1, C18:0, C18:1, C18:2 and C18:3 indicate palmitic, palmitoleic, stearic, oleic, linoleic and linolenic acids, respectively. (**d**) Fatty acid compositions in seeds from lines ‘XE3’ at four development stages. (**e**) Length of seeds from both lines at four development stages. (**f**) Width of seeds from both lines at four development stages. Error bars indicate standard deviations of three biological replicates. *Indicate significant differences of data between the two lines at the same developmental stage at the level of 0.05.

Sea buckthorn ‘SJ1’ and ‘XE3’ had previously been characterised as low and high oil content lines^34^, respectively. Here we explored oil accumulation, fatty acid composition and seed size across four developmental stages of the fruit from green (fruit swelling stage) to orange/red (fully matured stage). ‘XE3’ exhibited rapid accumulation (approximate 3.2-fold increase) of seed oil from 4.6% at G stage to 14.8% at G/Y stage (Fig. 1a,b), indicating a peak in oil accumulation between the early and the middle stages. Sea buckthorn seed oil has high concentrations of unsaturated fatty acids (>92%) such as palmitoleic (C16:1), oleic (C18:1), linoleic (C18:2) and linolenic (C18:3) acids (Fig. 1c,d). Six fatty acid components were measured in sea buckthorn seed oil. The relative content of palmitic (C16:0) and palmitoleic (C16:1) acids in line ‘SJ1’ (Fig. 1c) was higher than in line ‘XE3’ (Fig. 1d), but showed a decreasing trend with maturity in both lines. The relative contents of C18:1 and C18:2 first increased from G to Y/O stages and then decreased, and these two fatty acids contents in line ‘SJ1’ were lower than line ‘XE3’ from G/Y to O/R stages (Fig. 1c,d). The C18:2 levels peaked at the Y/O stage in both lines, while, C18:3 levels peaked in both lines at the G/Y stage.

Seed length in ‘SJ1’ was approximately 1.7-fold higher at the G/Y stage compared to the G stage. Interestingly seed lengths in ‘SJ1’ and ‘XE3’ decreased by 11.7% and 22.5%, respectively, from the G/Y to the O/R stage (Fig. 1e). The pattern for the width of seeds matched that of the length (Fig. 1f), indicating that growth of sea buckthorn seeds takes place mainly at early stages (G–G/Y). Thus, lipid biosynthesis and seed size in sea buckthorn is developmentally controlled.

### Identification of functional genes involved in lipid biosynthesis and seed size

**Figure 2:**
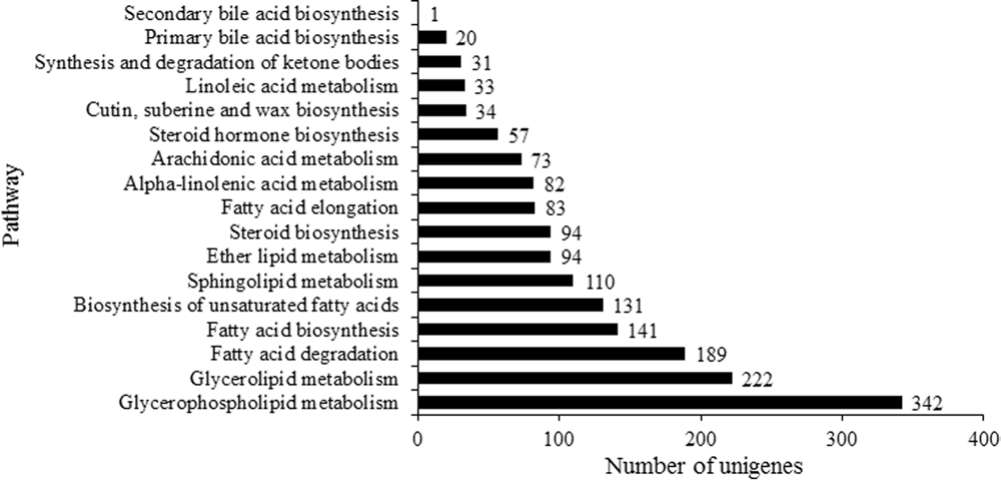
Distribution of annotated unigenes involved in lipid metabolism based on KEGG pathway analysis.

To explore unigenes involved in lipid biosynthesis and seed size, eight mRNA libraries from seeds extracted from four developmental stages (G, G/Y, Y/O, and O/R) were constructed by Illumina Hiseq2500, and 161,739,044 and 182,289,308 clean reads was generated for lines ‘SJ1’ and ‘XE3’ seeds, respectively (Supplementary Table S1). In total, 323,881 unigenes (≥200 bp) were assembled and used as reference for the discovery of pre-miRNA and miRNA sequences. Functional annotation revealed 79,413, 69,924,99,916 and 28,579 unigenes with alignments to the Nr (Non-redundant protein database), COG (Clusters of orthologous groups of protein), Swiss-Prot (Annotated protein sequence database) and KEGG (Kyoto encyclopedia of genes and genomes) databases, respectively. The GO (Gene ontology) database provides functional terms for genes across all species. In sea buckthorn transcriptome library, 167 and 520 functional unigenes were grouped into “developmental process” and “metabolic process”, respectively (Supplementary Table S2). In total, 3153 unigenes were assigned to “Lipid transport and metabolism” based on COG classifications according to phylogenetic relationships (Supplementary Table S2). We used KEGG pathway database with KAAS (KEGG Automatic Annotation Server) to predict the lipid biosynthesis network of sea buckthorn. The “lipid metabolism” category containing 1737 unigenes were grouped into 17 pathways (Fig. 2 and Supplementary Table S3). A maximum of 342 unigenes were involved in glycerophospholipid metabolism (ko00564) followed by 222 unigenes involved in glycerolipid metabolism (ko00561), and 141 unigenes involved in fatty acid biosynthesis (ko00061).

### Identification of known and novel miRNAs in developing sea buckthorn seeds

**Figure 3:**
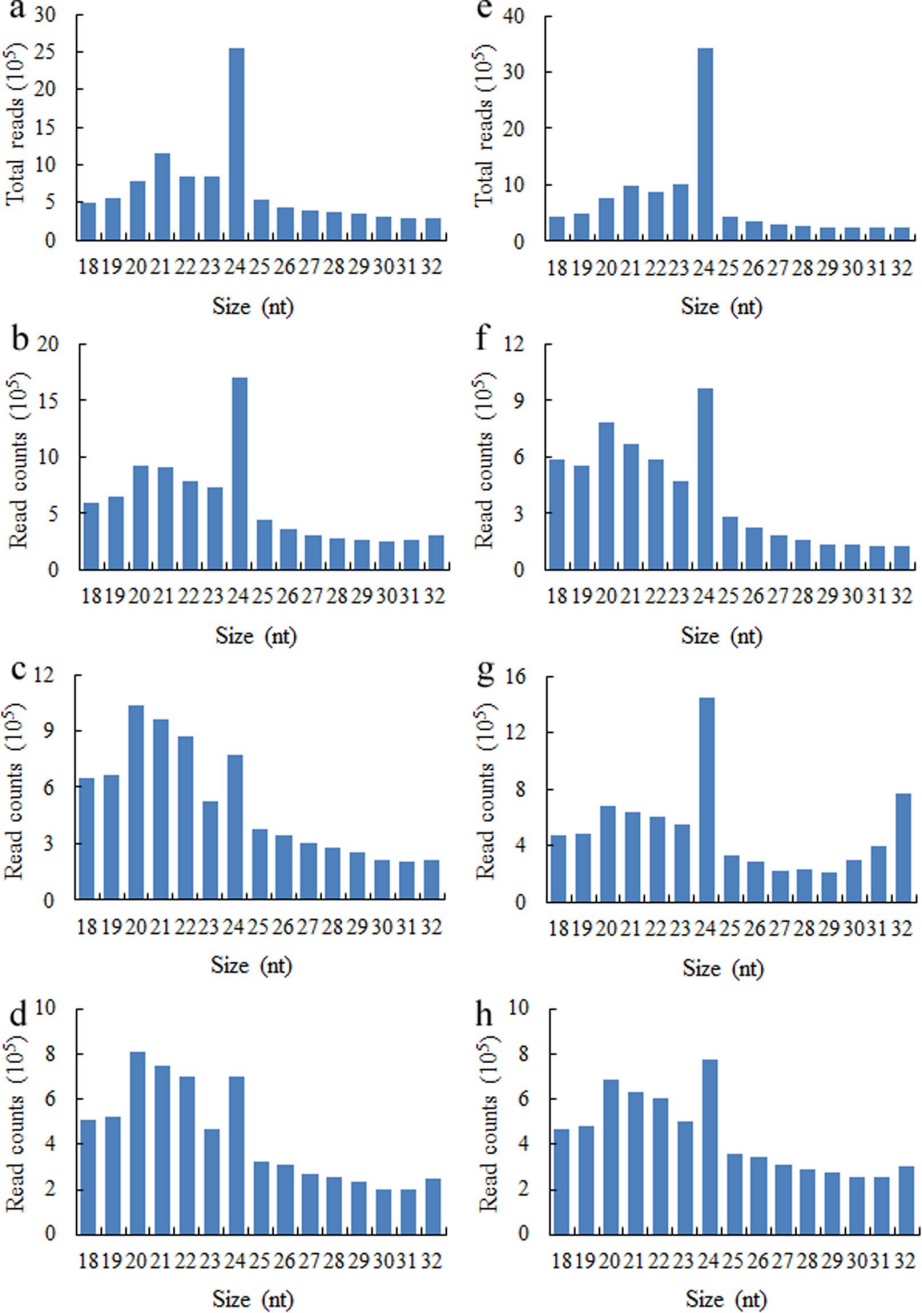
Length distribution of clean reads of eight small RNA libraries in sea buckthorn seeds. (**a**,**b**,**c** and **d**) Indicate clean reads of seeds from line ‘SJ1’ at four developmental stages G, G/Y, Y/O and O/R, respectively. (**e**,**f**,**g** and **h**) indicate clean reads of seeds from line ‘XE3’ at four developmental stages G, G/Y, Y/O and O/R, respectively. G, G/Y, Y/O and O/R indicate the developmental stage of the fruit as indicated by color green, green/yellow, yellow/orange and orange/red, respectively.

To identify miRNA-mediated regulation that may be involved in sea buckthorn lipid biosynthesis and seed size, eight sRNA libraries from the same eight samples as above were analyzed by Illumina Hiseq2500. Deep sequencing of all eight sRNA libraries yielded 33,184,369 and 30,410,875 clean reads in lines ‘SJ1’ and ‘XE3’, respectively (Table 1). Further quality evaluation of the sRNA sequencing data by the FastQC software identified high quality clean reads for sea buckthorn seed miRNAs (Supplementary Fig. S1). Within this dataset, 20 to 24 nts long sequences were the most abundant, with the 24 nts long sRNAs as most abundant in six libraries (G and G/Y stages in line ‘SJ1’, all four stages in line ‘XE3’) (Fig. 3a,b,e,f,g,h). This length distribution pattern of sRNAs in sea buckthorn is consistent with that in other plant species^13,35–37^. However, in the Y/O and O/R stages of line ‘SJ1’ seed datasets the number of 20 to 22 nts long sRNAs exceeded the number of 24 nts long sRNAs (Fig. 3c,d). The 20–22 nts plant sRNA mediate target gene cleavage or inhibition of protein translation, while the 24 nt sRNA class affects chromatin modelling of target genes^38^.

**Table 1:** Summary of sequencing data of eight sRNA libraries.

	Line ‘SJ1’ seeds					Line ‘XE3’ seeds		
	G	G/Y	Y/O	O/R	G	G/Y	Y/O	O/R
Raw reads*	14,468,271	14,339,275	13,300,277	10,891,810	14,025,713	11,691,931	13,401,496	11,479,969
Clean reads**	10,246,589	8,742,899	7,693,414	6,501,467	10,250,860	5,974,796	7,632,683	6,552,536
Q20 of clean read (%)	99.76	99.81	99.83	99.77	99.80	99.84	99.81	99.84
Q30 of clean read (%)	98.74	98.90	99.13	99.00	98.90	99.17	99.12	99.16
GC (%)	45.15	46.13	47.96	48.39	43.54	46.82	47.59	48.34

*The raw reads with lengths of 1 to 51 nts; **Clean reads with lengths of 18 to 32 nts; G, G/Y, Y/O and O/R indicate the developmental stage of the fruit as indicated by color green, green/yellow, yellow/orange and orange/red, respectively; Q20 and Q30 indicate the percentage of bases with Phred values >20 and >30, respectively; GC indicates the GC ratio of total base number.

Although these data suggest that different mechanisms may operate in the two lines during seed development, further studies will be required to clarify the significance of the abundant 20–22 nts class in line ‘SJ1’.

**Figure 4:**
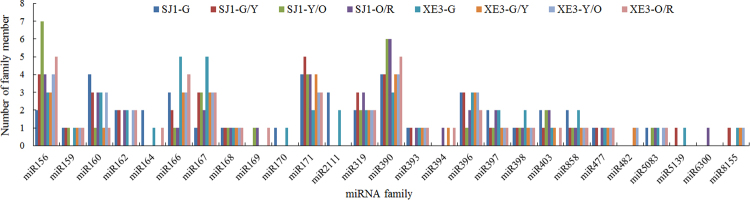
Distribution of known miRNA family size in sea buckthorn seed. SJ1-G, SJ1-G/Y, SJ1-Y/O and SJ1-O/R indicate the developmental stage of the line ‘SJ1’ fruit as indicated by color green, green/yellow, yellow/orange and orange/red, respectively; XE3-G, XE3-G/Y, XE3-Y/O and XE3-O/R indicate the developmental stage of the line ‘XE3’ fruit as indicated by color green, green/yellow, yellow/orange and orange/red, respectively.

The unique sRNA sequences were mapped against the miRBase sequence database (Release 20) allowing zero mismatches. A total of 137 known miRNAs sequences and pre-miRNAs were identified (Fig. 4 and Supplementary Table S4), of which 51 miRNAs were shared in both lines across the four seed developmental stages, and 47 and 39 miRNAs were identified to be development-specific in lines ‘SJ1’ and ‘XE3’, respectively. All miRNA family members and normalized reads are shown in Supplementary Table S5. All known miRNAs were clustered into 27 miRNA families (Fig. 4), among which 21 miRNA families (miR156, miR159, miR160, miR162, miR164, miR166, miR167, miR168, miR169, miR170, miR171, miR2111, miR319, miR390, miR393, miR394, miR396, miR397, miR398, miR403, and miR858) are highly conserved in *Arabidopsis*^15,39^, indicating that these conserved miRNAs may have the fundamental regulatory roles in developing sea buckthorn seed.

All of the non-annotated reads from the eight sRNA libraries were aligned with the miRBase sequence database (Release 20), and the miRDeep software was used to predict potential novel miRNAs^40^. A total of 264 potential novel miRNAs (designated as novelmiRNA-1 to novelmiRNA-264) were predicted (Supplementary Table S6). Four miRNAs (novelmiRNA-5, novelmiRNA-27, novelmiRNA-67, and novelmiRNA-74) in line ‘SJ1’, and six miRNAs (novelmiRNA-27, novelmiRNA-37, novelmiRNA-44, novelmiRNA-74, novelmiRNA-149, and novelmiRNA-261) in line ‘XE3’ were expressed at all four developmental stages (Supplementary Table S7). To determine miRNAs that were expressed specifically at any developmental stage, the significantly differentially expressed miRNAs were compared between libraries of two different developmental stages (16 pairwise comparison groups in Supplementary Table S8). A total of 177 down-regulated and 175 up-regulated miRNAs were identified among the 16 pairwise comparison groups (Table 2). These results suggest that miRNA-mediated regulatory mechanisms may have a significant role in sea buckthorn seed development and oil accumulation.

**Table 2:** Numbers of the significantly differential expression miRNAs for each of 16 pairwise comparison groups.

Pairwise comparison groups	Number of up-regulation miRNA	Number of down-regulation miRNA
SJ1-G *vs*. SJ1-G/Y	7	4
SJ1-G *vs*. SJ1-Y/O	5	0
SJ1-G *vs*. SJ1-O/R	7	9
SJ1-G/Y *vs*. SJ1-Y/O	5	4
SJ1-G/Y *vs*. SJ1-O/R	8	4
SJ1-Y/O *vs*. SJ1-O/R	2	9
XE3-G *vs*. XE3-G/Y	4	9
XE3-G *vs*. XE3-Y/O	9	6
XE3-G *vs*. XE3-O/R	9	13
XE3-G/Y *vs*. XE3-Y/O	20	15
XE3-G/Y *vs*. XE3-O/R	18	9
XE3-Y/O *vs*. XE3-O/R	14	7
SJ1-G *vs*. XE3-G	26	13
SJ1-G/Y *vs*. XE3-G/Y	16	20
SJ1-Y/O *vs*. XE3-Y/O	9	22
SJ1-O/R *vs*. XE3-O/R	18	31

SJ1-G, SJ1-G/Y, SJ1-Y/O and SJ1-O/R indicate the developmental stage of line ‘SJ1’ fruit as indicated by color green, green/yellow, yellow/orange and orange/red, respectively; XE3-G, XE3-G/Y, XE3-Y/O and XE3-O/R indicate the developmental stage of line ‘XE3’ fruit as indicated by color green, green/yellow, yellow/orange and orange/red, respectively.

### Identification of differentially expressed miRNAs and their target genes involved in lipid biosynthesis and seed size

Since the sea buckthorn genome has not as yet been published, we determined the *de novo* mRNA transcriptome of seeds from four developmental stages of ‘SJ1’ and ‘XE3’ for identification of miRNA target genes^41^. Using psRNATarget (plant miRNA target prediction server), 3074 gene targets were predicted. Some genes were targeted with multiple miRNA, leading to a total of 5594 putative miRNA-target interactions. The identified gene targets were aligned using BLASTX against the protein databases Nr, COG, and Uniprot, followed by GO and KEGG analysis (Supplementary Table S9).

The putative gene targets of known and novel miRNAs identified in this study were subjected to GO analysis to investigate gene ontology. One hundred and seven genes (targeted by 91 miRNAs) were involved in seven different cellular components, 197 genes (targeted by 148 miRNAs) took part in seven molecular functions, and 159 genes (targeted by 98 miRNAs) participated in nine biological processes. We followed the GO term “metabolic processes” containing 56 target genes (Supplementary Table S10); as examples acetyl-CoA carboxylase (*ACC*, c103701_g1_i1) and delta-9-desaturase (*Δ9D*, c119361_g2_i1) were grouped into subterm fatty acid biosynthetic process (GO: 0006633), while *GPD1* (c138230_g1_i3) was grouped into subterm carbohydrate metabolic process (GO: 0005975).

Based on the predicted miRNA-target interaction and functional annotation, we focussed on the miRNAs and their target genes involved in lipid biosynthesis. Four known (aly-miR170-5p, gma-miR168b, gma-miR164d, and zma-miR159i-3p) and 15 novel miRNAs were found to be involved in lipid biosynthesis (Table 3). Δ9D (c119361_g2_i1) targeted by gma-miR168b, *ACC* (c103701_g1_i1) targeted by novelmiRNA-108, glycerol-3-phosphate dehydrogenase (*GPD1*, c138230_g1_i3) targeted by novelmiRNA-23, diacylglycerol O-acyltransferase1 (*DGAT1*, c144982_g1_i2) targeted by novelmiRNA-58, and diacylglycerol O-acyltransferase 2 (*DGAT2*, c220405_g1_i1) targeted by novelmiRNA-191 were identified. The read counts of several novel miRNA, with potential target genes involved in lipid biosynthesis, were significantly higher in the low oil content line ‘SJ1’ as compared to the high oil content line ‘XE3’ (Supplementary Table S8). Based on these results it can be speculated that these miRNAs play an important role in regulating lipid biosynthesis.

**Table 3:** Predicted targets involved in lipid metabolism for miRNAs.

KEGG Pathway	miRNA name	Target ID	Annotation for targets	Gene name
Fatty acid biosynthesis	novelmiRNA-2	c141756_g2_i2	long-chain acyl-CoA synthetase	*ACSL*
	novelmiRNA-108	c103701_g1_i1	acetyl-CoA carboxylase carboxyl transferase	*ACC*
	novelmiRNA-110	c141756_g2_i2	long-chain acyl-CoA synthetase	*ACSL*
Fatty acid elongation	novelmiRNA-170	c168561_g1_i1	enoyl-CoA hydratase	*ECHS*
Fatty acid degradation	aly-miR170-5p	c60336_g1_i1	alcohol dehydrogenase	*adh*
	novelmiRNA-2	c141756_g2_i2	long-chain acyl-CoA synthetase	*ACSL*
	novelmiRNA-110	c141756_g2_i2	long-chain acyl-CoA synthetase	*ACSL*
	novelmiRNA-170	c168561_g1_i1	enoyl-CoA hydratase	*ECHS*
Biosynthesis of unsaturated fatty acids	gma-miR168b	c119361_g2_i1	delta-9-desaturase	*Δ9D*
	novelmiRNA-58	c145891_g1_i4	helix loop helix transcription factor	*HLH*
	novelmiRNA-77	c142283_g2_i1	helix loop helix transcription factor	*HLH*
Glycerolipid metabolism	gma-miR164d	c154991_g1_i1	dihydroxyacetone kinase	*DAK*
	novelmiRNA-11	c192054_g1_i1	phosphatidate phosphatase	*LPIN*
	novelmiRNA-23	c133634_g3_i5	phospholipid:diacylglycerol acyltransferase	*PDAT*
	novelmiRNA-58	c144982_g1_i2	diacylglycerol O-acyltransferase 1	*DGAT1*
	novelmiRNA-191	c220405_g1_i1	diacylglycerol O-acyltransferase 2	*DGAT2*
Glycero- phospholipid metabolism	novelmiRNA-11	c192054_g1_i1	phosphatidate phosphatase	*LPIN*
	novelmiRNA-23	c138230_g1_i3	glycerol-3-phosphate dehydrogenase	*GPD1*
	novelmiRNA-64	c176644_g1_i1	lysophospholipid hydrolase	*NTE*
Steroid biosynthesis	novelmiRNA-10	c81203_g2_i1	cycloartenol synthase	*CAS*
	novelmiRNA-58	c131674_g1_i1	sterol-4-alpha-methyl oxidase	*SMO2*
	novelmiRNA-58	c144982_g1_i3	sterol O-acyltransferase	*SOAT*
	novelmiRNA-179	c81203_g2_i1	cycloartenol synthase	*CAS*
	novelmiRNA-224	c137936_g1_i1	cycloartenol synthase	*CAS*
	novelmiRNA-224	c137936_g1_i2	lanosterol synthase	*LSS*
	novelmiRNA-232	c81203_g2_i1	cycloartenol synthase	*CAS*
Sphingolipid metabolism	zma-miR159i-3p	c262907_g1_i1	beta-galactosidase	*lacZ*
	novelmiRNA-23	c130447_g1_i3	neutral ceramidase	*ASAH2*
	novelmiRNA-108	c167130_g1_i1	sphingolipid delta-4 desaturase	*DEGS*
	novelmiRNA-151	c209847_g1_i1	beta-galactosidase	*GLB1*
	novelmiRNA-170	c199679_g1_i1	sphingomyelin phosphodiesterase 2	*SMPD2*

Many transcription factors play an essential role in controlling seed size^26,42^, however, the transcription factor regulatory network controlling seed size in woody oil crops remains largely unknown. Previous studies showed that ARF, MYB, CNR (cell number regulator) transcription factors and the *MED* gene play key role in controlling seed development and seed size in model plants^28–31,43^. In developing sea buckthorn seed, the gene targets of several miRNAs encode ARF, MYB, and CNR transcription factors (Table 4), including *ARF2* (c88882_g1_i1 and c145594_g1_i3) targeted by gma-miR164d; *ARF18* (c145777_g1_i1) targeted by gma-miR160b, gma-miR160c, gma-miR160d, and gma-miR160e; *MYB* (c133448_g2_i1) targeted by aly-miR166e-5p; and *CN*R13 (c37818_g1_i1) targeted by novelmiRNA-60, novelmiRNA-86, novelmiRNA-93, and novelmiRNA-196 (Table 4). CNR transcription factor are considered as general regulators of plant cell number and organ size. In sea buckthorn seed, *MED12* (c180446_g1_i1), *MED16* (c172958_g1_i1), and *MED30* (c129331_g2_i1) were targeted by novelmiRNA-98, novelmiRNA-28, and novelmiRNA-23, respectively. Whether these genes control seed size in sea buckthorn remain to be determined.

**Table 4:** Predicted targets involved in seed size for miRNAs.

miRNA name	Target ID	Annotation for targets	Gene name
gma-miR164d	c88882_g1_i1	auxin response factor 2	*ARF2*
gma-miR164d	c145594_g1_i3	auxin response factor 2	*ARF2*
novelmiRNA-167	c146624_g3_i1	auxin response factor 2	*ARF2*
gma-miR160d	c145777_g1_i1	auxin response factor 18	*ARF18*
gma-miR160b	c145777_g1_i1	auxin response factor 18	*ARF18*
gma-miR160c	c145777_g1_i1	auxin response factor 18	*ARF18*
gma-miR160e	c145777_g1_i1	auxin response factor 18	*ARF18*
novelmiRNA-204	c145777_g1_i1	auxin response factor 18	*ARF18*
aly-miR166e-5p	c133448_g2_i1	MYB domain-containing protein	*MYB*
novelmiRNA-48	c146969_g1_i2	Myb-like protein L	*MYB*
novelmiRNA-76	c146969_g1_i2	Myb-like protein L	*MYB*
novelmiRNA-122	c146969_g1_i2	Myb-like protein L	*MYB*
novelmiRNA-128	c146969_g1_i2	Myb-like protein L	*MYB*
novelmiRNA-174	c147552_g2_i2	Myb-related protein	*MYB*
novelmiRNA-201	c146969_g1_i2	Myb-like protein L	*MYB*
novelmiRNA-234	c146969_g1_i2	Myb-like protein L	*MYB*
novelmiRNA-23	c129331_g2_i1	mediator of RNA polymerase II transcription subunit 30	*MED30*
novelmiRNA-28	c172958_g1_i1	mediator of RNA polymerase II transcription subunit 16	*MED16*
novelmiRNA-98	c180446_g1_i1	mediator of RNA polymerase II transcription subunit 12	*MED12*
novelmiRNA-60	c37818_g1_i1	cell number regulator 13	*CNR13*
novelmiRNA-86	c37818_g1_i1	cell number regulator 13	*CNR13*
novelmiRNA-93	c37818_g1_i1	cell number regulator 13	*CNR13*
novelmiRNA-196	c37818_g1_i1	cell number regulator 13	*CNR13*

### qRT-PCR validation of miRNAs and corresponding target genes

**Figure 5:**
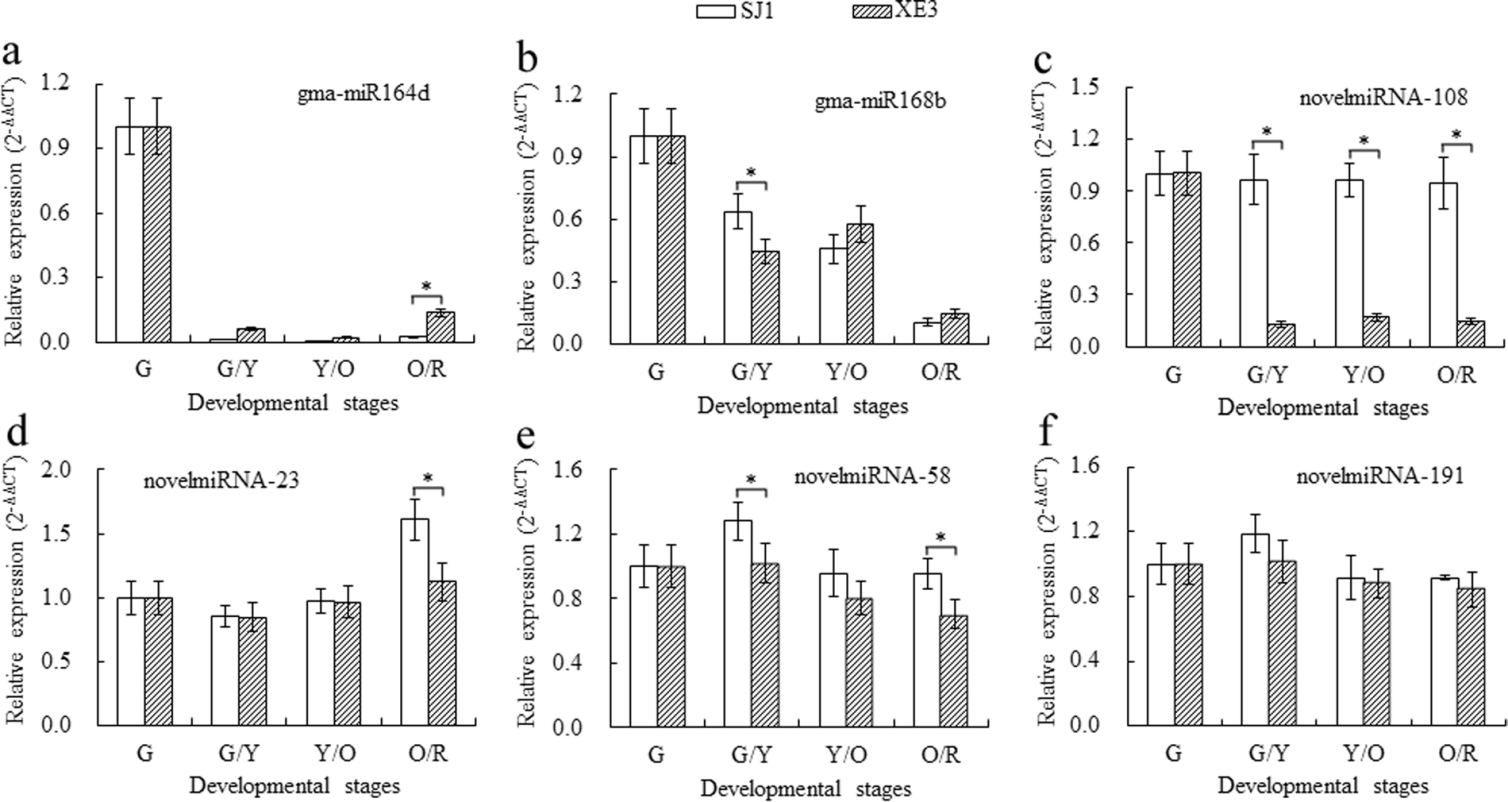
qRT-PCR validation of select miRNAs putatively related to lipid biosynthesis and seed size in sea buckthorn. (**a**) Gma-miR164d expression. (**b**) Gma-miR168b expression. (**c**) NovelmiRNA-108 expression. (**d**) NovelmiRNA-23 expression. (**e**) NovelmiRNA-58 expression. (**f**) NovelmiRNA-191 expression. G, G/Y, Y/O and O/R indicate the developmental stage of the fruit as indicated by color green, green/yellow, yellow/orange and orange/red, respectively. *Indicate significant differences of gene relative expression level between the two lines at the same developmental stages, at the level of 0.05.

**Figure 6:**
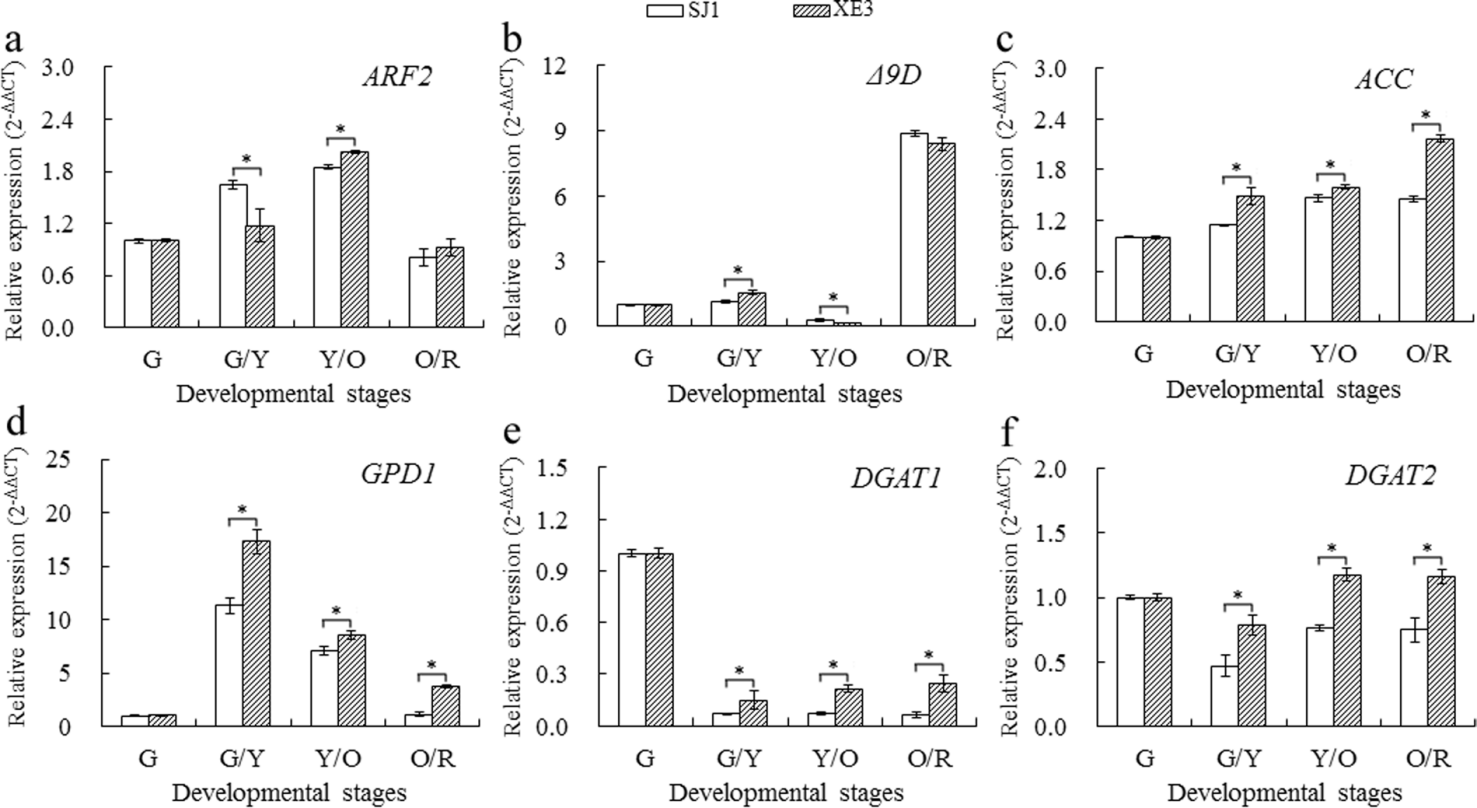
qRT-PCR validation of target genes related to lipid biosynthesis and seed size in sea buckthorn. (**a**) *ARF2* expression. (**b**) *Δ9D* expression. (**c**) *GPD1* expression. (**d**) *DGAT1* expression. (**e**) *DGAT2* expression. (**f**) NovelmiRNA-191 expression. G, G/Y, Y/O and O/R indicate the developmental stage of the fruit as indicated by color green, green/yellow, yellow/orange and orange/red, respectively. *Indicate significant differences of gene relative expression level between the two lines at the same developmental stages, at the level of 0.05.

An integrated analysis of mRNA and miRNA transcriptome and qRT-PCR identified some miRNAs and their targets (miR164d-ARF2, miR168b-Δ9D, novelmiRNA-108-ACC, novelmiRNA-23-GPD1, novelmiRNA-58-DGAT1, and novelmiRNA-191-DGAT2) potentially involved in seed size and lipid biosynthesis (Figs 5 and 6). The qRT-PCR data of miRNAs matched the expression profiles obtained by sRNA sequencing of the G, G/Y, Y/O and O/R libraries, and the qRT-PCR data of the corresponding target gene showed a trend opposite to that of miRNA expression (Fig. 6). Relative expression levels of gma-miR164d, gma-miR168b, and novelmiRNA-108 decreased in both lines from G to G/Y stages (Fig. 5a,b,c), but gma-miR164d and gma-miR168b expression levels were up-regulated at O/R and Y/O stages, respectively, in seed of line ‘XE3’. By contrast, *ARF2* (c145594_g1_i3), a target of gma-miR164d, was up-regulated from G to Y/O stages and then down-regulated at O/R stage (Fig. 6a). *Δ9D* gene (c119361_g2_i1) targeted by gma-miR168b was up-regulated at G/Y stage and then down-regulated at Y/O stage followed by a sharp increase at the O/R stage. *Δ9D* expression in ‘SJ1’ seed was higher than in ‘XE3’ seed (Fig. 6b). *ACC* (c103701_g1_i1) targeted by novelmiRNA-108 was up-regulated in both lines with higher expression in ‘XE3’ seed (Fig. 6c).

The novelmiRNA-23 was first down-regulated at G/Y stage and then up-regulated from Y/O to O/R stages in both lines (Fig. 5d). The novelmiRNA-58 and novelmiRNA-191 were first up-regulated and then down-regulated (Fig. 5e,f), and their expression levels in ‘SJ1’ seed were higher than in ‘XE3’ from G/Y to O/R stages. On the contrary, *GPD1* (c138230_g1_i3) targeted by novelmiRNA-23 first increased at G/Y stage and then declined from Y/O to O/R stages (Fig. 6d). *DGAT1* (c144982_g1_i2) and *DGAT2* (c220405_g1_i1) targeted by novelmiRNA-58 and novelmiRNA-191, respectively, first declined at G/Y stage and then increased from Y/O to O/R stages. The expression levels of *GPD1*, *DGAT1*, and *DGAT2* in ‘XE3’ seeds were significantly higher than in ‘SJ1’ seeds from G/Y to O/R stages (Fig. 6e,f).

### Regulation of *Δ9D* by miR168b, as determined by luciferase activity assays

**Figure 7:**
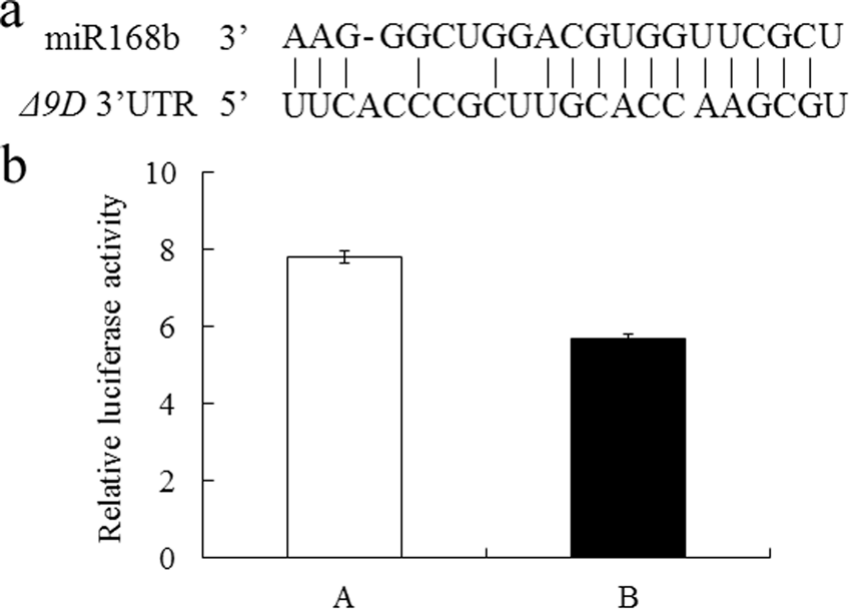
The validation of miR168b and target *Δ9D*. (**a**) Prediction of the binding sites of miR168b in *Δ9D* mRNA using psRNATarget. (**b**) Effect of miR168b expression on luciferase activity in transfected cells: (A) pCDNA3.1 + pmirGLO-*Δ9D*, (B) pCDNA3.1-miR168b + pmirGLO-*Δ9D*. Data are represented as the mean ± standard deviation (SD) from three independent experiments.

Target prediction analysis server psRNAtarget was used to assess the complementarity between miR168b and the target site. miR168b was predicted to have the potential to target *Δ9D* 3′UTR (Fig. 7a). Luciferase activity in 293 cells co-transfected with the miR168b recombinant expression vector and the expression vector containing the 3′UTR of *Δ9D* fused with the reporter gene was decreased by nearly 26.9% (*p* < 0.05) compared to that in the control group (Fig. 7b). These results indicate that *Δ9D* is one of the target genes of miR168b.

## Discussion

Sea buckthorn (*Hippophae* L.) is a nutritionally and ecologically important woody plant known for the unique composition of its seed and fruit oil^1^. Although genes related to oil biosynthesis have been identified in sea buckthorn in previous^2,44^ and present studies, the regulatory mechanism of sea buckthorn oil accumulation is poorly understood. To explore if miRNA-mediated post-transcriptional regulation is involved in controlling oil accumulation and seed size in sea buckthorn, we analyzed the dynamic patterns of oil content, fatty acids composition, and seed size of developing seeds of lines ‘SJ1’ and ‘XE3’ (Fig. 1). Line ‘SJ1’ has low seed oil content and bigger seeds, and line ‘XE3’ has high seed oil content and smaller seeds^34^. We carried out comparative deep miRNA transcriptomic analysis in the two lines at four developmental stages of seeds, which generated 33,184,369 and 30,410,875 clean reads for lines ‘SJ1’ and ‘XE3’, respectively (Table 1). Bioinformatics analysis identified a total of 137 known and 264 novel miRNAs in developing sea buckthorn seeds (Supplementary Table S4 and Supplementary Table S6). Nineteen (four known and 15 novel) and 22 (six known and 16 novel) miRNAs were found to be involved in lipid biosynthesis and seed size, respectively, suggesting that miRNAs regulate oil accumulation and seed size in sea buckthorn seeds.

### miRNA and their target genes involved in fatty acid biosynthesis in sea buckthorn

**Figure 8:**
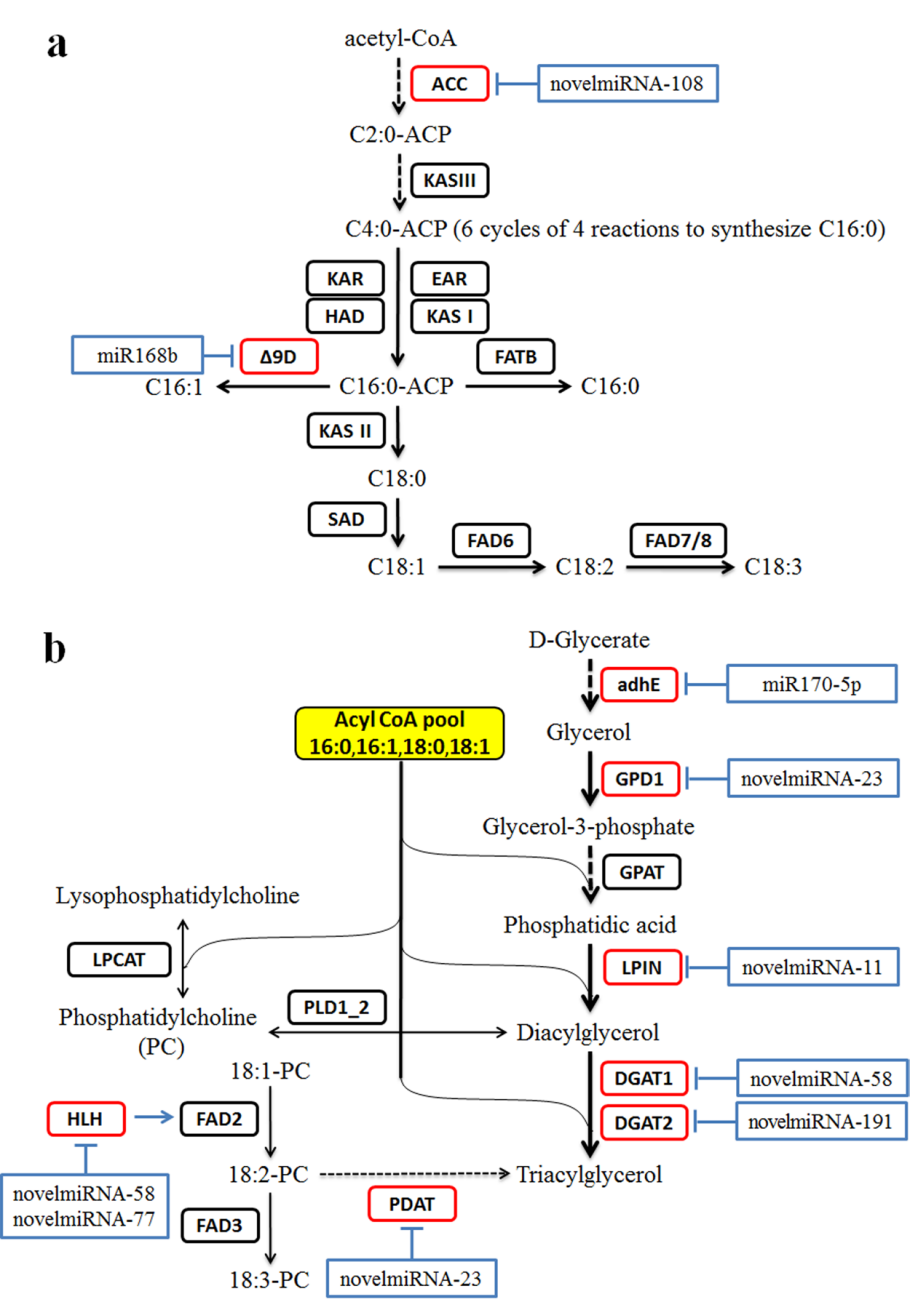
Sea buckthorn sequences associated with fatty acid (**a**) and TAG (**b**) biosynthetsis pathways. The miRNAs and its putative target genes are shown in blue and red boxes, respectively. Acetyl-CoA carboxylase (*ACC*); 3-oxoacyl-ACP synthase I/II/III (*KAS I/II/III*); 3-oxoacyl-ACP reductase (*KAR*); enoyl-acyl-ACP reductase (*EAR*); 3-hydroxyacyl-ACP dehydratase (*HAD*); delta-9-desaturase (*Δ9D*); fatty acyl-ACP thioesterases B (*FATB*); stearoyl-ACP desaturase (*SAD*); fatty acid desaturase (*FAD*); aldehyde dehydrogenases (*adhE*), glycerol-3-phosphate dehydrogenase (*GPD1*); glycerol-3-phosphate acyltransferase (*GPAT*); diacylglycerol O-acyltransferase (*DGAT*); phosphatidate phosphatase (*LPIN*); lysophosphatidylcholine acyltransferase (*LPCAT*); phospholipase D (*PLD1_2*); helix loop helix (*HLH*); phospholipid diacylglycerol acyltransferase (*PDAT*).

Eight miRNAs associated with fatty acid metabolic pathways were identified (Table 3). The expression of *Δ9D* gene that is involved in the conversion of C16:0 to C16:1^44^ (Fig. 8a) was up-regulated at G/Y stage, down-regulated at Y/O stage, and sharply increased at O/R stage (Fig. 6b). *Δ9D* is a direct target of gma-miR168b, and its expression was significantly down-regulated in gma-miR168b transduced cells (Fig. 7b). As would be expected the expression trends of *Δ9D* and gma-miR168b were opposite of each other (Figs 5b and 6b), thus confirming that the gma-miR168b-target *Δ9D* relationship in conserved in sea buckthorn. Interestingly, while the expression of *Δ9D* was sharply up-regulated at the O/R stage, the C16:1 levels in both lines remained very low (Fig. 1c,d). This could be due to further regulation of *Δ9D* at the post-transcriptional level or lack of enough precursor C16:0-ACP. The down-regulation of *Δ9D* at Y/O stage was coordinated with high expressions of ketoacyl-ACP reductase (*KAR*) and ketoacyl-ACP synthase II (*KAS II*) and increased and decreased levels of C18:0 and C16:1, respectively (Fig. 8a), indicating higher precursor availability for the synthesis of C18 unsaturated fatty acids^45^. It is possible that inhibition of *Δ9D* by gma-miR168b in sea buckthorn seed directs conversion of C16:0 to higher levels of C18:2 and C18:3. It would be interesting to study the regulation of miR168b in sea buckthorn fruit pulp which, unlike the seed, contains high levels of C16:1 that is important for human health, but is also a valuable renewable source for industrial chemicals and biodiesel^46^. miR168 is also stress- and abscisic acid- inducible^47^. Furthermore, in mice fed on rice grain containing high levels of miR168a, the rice miR168a could bind to mRNA encoding human/mouse low-density lipoprotein receptor adapter protein 1 (LDLRAP1), thereby reducing its expression in the liver and increasing LDL levels in mouse plasma^48^.

Expression of novelmiRNA-108 increased steadily with seed development in line ‘SJ1’, but declined sharply at the G/Y stage in ‘XE3’, which had significantly lower levels of this miRNA than in ‘SJ1’ (Fig. 5c). One of the putative targets of this miRNA is the *ACC* gene, which encodes the first committed enzyme that controls the flux of carbon into fatty acids^49^ (Fig. 8a). *ACC* was up-regulated in both lines from G/Y to O/R stages and its expression was significantly higher in ‘XE3’ as compared to ‘SJ1’ (Fig. 6c), which agree with the higher oil content in ‘XE3’ (Fig. 1b).

The putative target of novelmiRNA-58 is an HLH transcription factor. This protein binds as a homodimers or as heterodimers to specific target sequences in the *FAD2* gene promoter^25^. FAD2 catalyzes the first extra-plastidial desaturation in plants, converting oleic acid to linoleic acid. The expressions of novelmiRNA-58 was higher in ‘SJ1’ than in ‘XE3’, and it increased at the G/Y stage in ‘SJ1’ and then decreased from G/Y to O/R stages (Fig. 5e). Interestingly, the level of C18:2 in ‘SJ1’ seeds were slightly lower than in ‘XE3’ seeds from G/Y to O/R stages (Fig. 1c,d). Further validation in required of the roles of novelmiRNA-108 and novelmiRNA-58 in fatty acids biosynthesis.

### The TAG biosynthesis miRNA and their targets in sea buckthorn

A primary substrate for TAG biosynthesis is glycerol-3-phosphate (G3P) and its levels directly limit TAG biosynthesis in seeds^50^. G3P can be catalysed by glycerol-3-phosphate dehydrogenase (GPD1) from glycerol^51^ (Fig. 8b). GPD1, a rate-limiting enzyme of lipid synthesis^52^, plays a key role in carbohydrate and lipid metabolism^53^. A twofold increase in GPD activity led to a three to four -fold increase in the level of G3P in transgenic oil-seed rape, resulting in a 40% increase in the final oil content of the seed^50^.

In the Kennedy pathway, the enzyme glycerol-3-phosphate acyltransferase (*GPAT*) utilizes G3P and acyl-CoA as substrates to form lysophosphatidic acid (*Lyso-PA*), which is acylated by 1-acyl-sn-glycerol-3-phosphate acyltransferase (*LPAAT*) to form phosphatidic acid (PA). Next, PA is dephosphorylated by phosphatidate phosphatase (*LPIN*) to form diacylglycerol (DAG), which is finally converted to TAG by diacylglycerol O-acyltransferase (*DGAT1* and *DGAT2*)^44,49^ (Fig. 8b). It is well known that the DGAT enzyme catalyzes a rate-limiting reaction in TAG bioassembly, and *DGAT1* and *DGAT2* genes are responsible for the progress of this reaction^54^. Seed-specific overexpression of *AtDGAT1* increased the seed oil content by up to 8.3% in transgenic *Brassica juncea* as compared to wild type plants^55^. The expression patterns of both *XsDGAT1* and *XsDGAT2* correlated with oil accumulation in developing *Xanthoceras sorbifolia* embryos, and overexpression of these genes increased total seed oil content in transgenic plants as compared to wild-type plants^54^. Furthermore, we revealed in sea buckthorn that the high coordinated expression of source ‘*GPD1*’ and sink ‘*DGAT1* and *DGAT2*’ genes results in oil accumulation in the pulp^56,57^.

In the present study, sea buckthorn *DGAT1* and *DGAT2* were targeted by novelmiRNA-58 and novelmiRNA-191, respectively. The expression of novelmiRNA-58 and novelmiRNA-191 peaked at the G/Y stage in both lines (Fig. 5e,f) and then decreased. The target genes *DGAT1* and *DGAT2* showed a trend opposite to those of miRNAs. Also *DGAT1* and *DGAT2* expression was lower in ‘SJ1’ as compared to ‘XE3’ (Fig. 6e,f), while the expression of novelmiRNA-58 and novelmiRNA-191 was always higher in ‘SJ1’ compared to ‘XE3’ (Fig. 5e,f). These results suggest that *DGAT1* and *DGAT2* genes are regulated by the novel miRNAs but further validation is required.

We identified the putative target of novelmiRNA-23 as *GPD1*, which was expressed at higher levels in ‘XE3’ as compared to ‘SJ1’, and in both lines it was first up-regulated at G/Y stage and then down-regulated (Fig. 6d). The gene expression changes correlated with oil content changes in both lines (Fig. 1b), and, as would be expected, were opposite to the expression trends of the miRNAs (Fig. 5d). In addition to establishing a putative miRNA-target gene relationship, these results clearly indicate that most TAG biosynthesis occurrs at the early to -mid stage of sea buckthorn seed development (Fig. 1b). Earlier *GPD1* was identified as the target of jcu_MIR403 in *Jatropha*, and of oco-miR044 in *Oryza coarctata* seeds, and *GPD1* expression was correlated to increased carbon flux and TAG biosynthesis^58,59^. Future studies will reveal how important the role of novelmiRNA-23 is in regulating *GPD1* and consequently TAG biosynthesis in sea buckthorn seeds.

### The seed size miRNAs and their targets in sea buckthorn

ARFs are transcription factors involved in auxin signal transduction during many stages of plant growth development via regulation of auxin response genes^26^. The crucial roles of ARFs in distinct biological processes and tissues are well understood in *Arabidopsis*. For example, mutation in *ARF5* impairs the initiation of the body axis. Multiple ARF family members act as targets of conserved plant miRNA families including miR160, miR167 and miR390, and ARF regulate miRNA expression^60,61^. For example, *ARF10, ARF16* and *ARF17* transcript levels were highly increased in the miR160 *foc* mutant during early embryogenesis, and miR167, which targets *ARF6* and *ARF8*, is preferentially expressed in rice seed, suggesting its involvement during late embryogenesis (seed maturation)^26^.

In this study, gma-miR164d was predicted to target *ARF2*, which is a repressor of cell division and organ growth, and determines the final size of the seed^62^. gma-miR164d showed very high expression at G stage, then relatively low expression at G/Y and Y/O stages, and then again up-regulated at the O/R stage (Fig. 5a). As would be expected, *ARF2* was up-regulated during the G/Y and Y/O stages (Fig. 6a), when the length and width of seeds decreased (Fig. 1e,f). While the change in seed size cannot be fully explained by the expression patterns of miR164d (Fig. 1e,f), the possibility of regulation of *ARF2* by miR164d cannot be ruled out at all seed developmental stages.

Two key determinants of organ size are cell number and cell size, and altering either one may affect the plant organ size. CNR and MED are considered as general regulators of plant cell number, final organ size, and fruit growth^28,43^. *CNR1* reduced maize ear length and overall plant size when overexpressed, and the ear length increased when its expression was silenced^28^. Overexpression of *MED25* produced small organs owing to decreases in both cell number and cell size in *Arabidopsis*^43^. In this study, *CNR13*, *MED12*, *MED16*, and *MED30* were targeted by novelmiRNA-60, novelmiRNA-98, novelmiRNA-28, and novelmiRNA-23, respectively, indicating that several novel miRNAs may be involved in controlling seed size in sea buckthorn.

In conclusion, conserved and novel miRNAs were identified in two sea buckthorn lines. The putative identities of target genes of some miRNAs indicate that lipid biosynthesis and seed size, among other physiological processes, may be regulated by miRNAs in sea buckthorn.

## Materials and Methods

### Plant materials

Sea buckthorn lines ‘SJ1’ and ‘XE3’ belonged to *H. rhamnoides* ssp. *mongolica* grew at the Institute of Berries, Heilongjiang Academy of Agricultural Sciences in Suiling county, Heilongjiang Province, China (47°14′12.3″ northern latitude, 127°05′39.9″ east longitude). The orchard had a mean annual rainfall of 570.6 mm, mean annual temperature of 2.0 °C, mean annual evaporation capacity of 1242.5 mm and effective accumulative temperature of 2460.4 °C^63^. Line ‘SJ1’ was selected from seedlings of cultivar ‘Wulangemu’ (*H. rhamnoides s*sp. *mongolica*) in 1990s, and line ‘XE3’ was selected from seedlings of Russia cultivars (*H. rhamnoides* ssp. *mongolica*) in 2000s. Only one pollinate tree was cultivar ‘Wucixiong’ (ssp. *mongolica*.). The three trees of line ‘SJ1’ for collecting fruits were all cutting seedlings for three biological repetitions; the same was for line ‘XE3’. So the lines are stable and conserved over the generation. The molecular marker-based genetic similarity of these two lines is 0.761^64^. Fruits of both lines at four developmental stages described as green (G), green/yellow (G/Y), yellow/orange (Y/O) and orange/red (O/R)^2^ were harvested in 2015. The fruits of line ‘SJ1’ were collected on 25 June (G), 17 July (G/Y), 8 August (Y/O) and 30 August (O/R); the fruits of line ‘XE3’ were collected on 6 July (G), 28 July (G/Y), 19 August (Y/O) and 10 September (O/R). The samples were immediately frozen in liquid nitrogen and stored at −80 °C.

### Oil content and fatty acid composition analysis

Sea buckthorn seed oil was isolated using a methanol-chloroform extraction procedure^65,66^. Seed sample powder (300 mg) was homogenised in methanol (2 ml) for 1 min, and after adding chloroform (4 ml) homogenization was continued for a further 2 min. The mixtures were sonicated in an ultrasonic bath for 30 min, centrifuged and filtered. The solid residues were re-suspended in chloroform/methanol (2:1, v/v, 4 ml) and homogenised again for 3 min and filtered. The volume of 1 ml of 0.88% KCl solution was added to the combined filtrates, and the mixtures were mixed thoroughly by vortexing and then centrifuged. The lower phase containing the purified oils was collected and evaporated to dryness under nitrogen.

Fatty acid composition was determined as fatty acid methyl esters (FAMEs) based on the boron trifluoride in methanol catalysis^67^. GC–TOF/MS analysis of FAMEs was performed on a Clarus 680 GC coupled with AxION iQT TOF/MS system (PerkinElmer, Shelton, USA). The system was equipped with Agilent J&W DB-23 capillary column (60 m × 0.25 mm × 0.25 μm). Fatty acid composition was measured and expressed as weight percentage of each fatty acid to the total fatty acids according to our previous study^45^. The analyses were conducted in three replicates.

### mRNA and sRNA library construction and Illumina high-throughput sequencing

Total RNA was isolated from four developmental stages of lines ‘SJ1’ and ‘XE3’ seeds, using TRIzol RNA Extraction Kit (Invitrogen, Carlsbad, CA, USA). Total RNA was quantified and qualified by Agilent 2100 Bioanalyzer (1.9 < A260/A280 < 2.1, 2.0 < A260/A230 < 2.5 and RNA Integrity Number value ≥ 8.0). Next generation sequencing libraries were constructed according to the manufacturer’s protocol (NEBNext® Ultra™ RNA Library Prep Kit for Illumina). Enrichment of mRNA, fragment interruption, addition of adapters, size selection and PCR amplification were performed by GENEWIZ Inc. (Suzhou, China), and RNA-Seq was conducted by Illumina HiSeq2500 System. *De novo* assembly of the clean reads after removal of ambiguous nucleotides (N) and low-quality bases of raw reads based on Q20, Q30, N and GC parentages, was performed using Trinity software r2013-02-25^68^. All unigenes were aligned using Blastx algorithms (E-value ≤ 10^−5^) to identify homologous genes, and to the Nr, COG, Swiss-Prot and KEGG databases^69^. The unigenes were mapped to the KEGG metabolic pathway database to elucidate the complex biological behaviors of unigenes using KAAS^70^. Orthologous gene products were classified and the functions of unigenes was predicted using the COG database. GO classifications of unigenes were obtained using WEGO software^71^ after annotation by the Blast2GO program^72^ to elucidate the distribution of gene functions.

To identify miRNA involved in regulating lipid biosynthesis and seed size in sea buckthorn seeds, the RNA samples from four developmental stages of lines ‘SJ1’ and ‘XE3’ were used for small RNA sequences. Eight cDNA libraries were constructed according to the manufacturer’s protocol (NEBNext® Multiplex Small RNA Library Prep Set Kit for Illumina). The libraries were multiplexed and loaded on an Illumina HiSeq2500 instrument according to manufacturer’s instructions. The sequences were processed and analyzed by GENEWIZ Inc. (Suzhou, China).

### Identification and analysis of known and novel miRNAs

The overall procedure for analyzing sRNA libraries is shown in Supplementary Fig. S1. All low-quality reads were removed, and 5′ and 3′ adapter sequences were trimmed using Genome Analyzer Pipeline v1.9. The remaining low -quality reads with ‘n’ were removed using Trimmomatic v0.3. Sequences shorter than 18 nts and longer than 32 nts were excluded from further analysis. Small RNAs were identified by mapping with miRDeep2 software and excluded from further miRNA predictions and analyses.

To identify conserved plant miRNAs in sea buckthorn, sRNA sequences were aligned with known plant miRNAs (Viridiplantae) in the miRBase database (Release 20) using miRDeep2 software. Complete alignment of the sequences was required and zero mismatches were allowed. To search for novel miRNAs, small RNA sequences were matched against assembled mRNA-seq contigs using the MiRanda software. As miRNA precursors have a characteristic hairpin structure, the next step to select candidate sequences was secondary structure analysis using MiRDeep2. Stem-loop structures should have the miRNA sequence at one arm of the stem and a corresponding antisense sequence at the opposite arm. Finally, precursor candidate sequences were checked using the BLASTn algorithm from the miRBase (www.miRBase.org) and NCBI databases.

For the frequency analysis of all identified miRNAs, sRNA reads were aligned in MiRDeep2 software. As reference, we used both previously annotated pre-miRNAs from miRBase and the putative pre-miRNAs identified in this work. The read counts of identified miRNAs in eight libraries were normalized as transcripts per million (TPM) according to the formula: Normalized expression = actual miRNA count/total count of clean reads × 1,000,000^14^. To assess whether the miRNA was differentially expressed, we independently used the R package EdgeR^73^. We considered miRNAs to be significantly differential expression if they had *p* value ≤ 0.05 and log_2_ (fold change) ≥2.

### Prediction of miRNA targets

Prediction of target genes of novel miRNAs was performed against assembled RNA-seq contigs using psRNAtarget^74^, with the default parameters and a maximum expectation value of 4 (number of mismatches allowed). Function of each targeted gene was identified based on in-house sea buckthorn fruits mRNA-seq data; this analysis was conducted using the blast2GO v2.3.5 software^75^. Annotation was improved by analyzing conserved domains/families using the InterProScan tool. Orientation of the transcripts was obtained from BLAST annotations.

### qRT-PCR analysis of miRNAs and their target genes

The RNA samples used for qRT-PCR analysis were the same as those for mRNA and miRNA sequence experiments. RNA was extracted as described under sRNA library preparation. First-strand cDNA synthesis was carried out using the Mir-X^TM^ miRNA first-strand cDNA synthesis kit (TaKaRa), and qRT-PCR of miRNAs was performed using the Mir-X^TM^ miRNA qRT-PCR SYBR kit (TaKaRa) and miRNA-specific primers in an ABI 7500 Real-Time PCR system (Applied Biosystems, Foster, USA). Small nuclear RNA U6 was used as an internal control. All samples included three technical repetitions. The primer sequences are shown in Supplementary Table S11.

Predicted target genes were validated by qRT-PCR using specific primers designed with Primer Premier 5.0. The first strand cDNA was synthesized from the RNAs using a PrimeScript^TM^ RT reagent Kit with gDNA Eraser (TaKaRa, Dalian, China). qRT-PCR was performed in an ABI 7500 Real-Time PCR system (Applied Biosystems, Foster, USA) using the SYBR Premix Ex Taq^TM^ II Kit (TaKaRa, Dalian, China) according to the manufacturer’s instructions. The relative gene expression for qRT-PCR data was calculated by the 2^−ΔΔCt^ method. All of the analyzed target genes were tested with three replicates. The primer sequences are shown in Supplementary Table S12.

### Dual-luciferase reporter assay

Fragments from the 3′UTR of Δ9D containing the predicted binding sequences for miR168b were amplified and sub-cloned into pmirGLO luciferase promoter vector. The pCDNA3.1 plasmid was used as the template vector. The fragment containing the nucleotide sequences of precursor of the miR168b were cloned into the vector to construct the recombinant vector expressing miR168b pCDNA3.1 as described earlier. The pmirGLO vector containing 3′UTR of Δ9D were co-transfected with pCDNA3.1 or pCDNA3.1 containing pre-miR168b using lipofectamine 2000 (Invitrogen, Carlsbad, CA, USA) according to the manufacturer’s protocol and previous report^76–78^. Forty-eight h after treatment, the expressed luciferase firefly and renilla activity was measured using a luciferase reporter assay kit (BioVision, Inc., CA, USA). Renilla was used as a transfection control.

## Electronic supplementary material

Supplementary information

Supplementary Table S1

Supplementary Table S2

Supplementary Table S3

Supplementary Table S4

Supplementary Table S5

Supplementary Table S6

Supplementary Table S7

Supplementary Table S8

Supplementary Table S9

Supplementary Table S10

Supplementary Table S11

Supplementary Table S12
