# Supplementary material for: Identification of microRNAs involved in lipid biosynthesis and seed size in developing sea buckthorn seeds using high-throughput sequencing
Source: Sci Rep. 2018 Mar 5;8:4022. doi: 10.1038/s41598-018-22464-w (PMC5838164; doi:10.1038/s41598-018-22464-w)
Supplement: Supplementary file 1 — Supplementary information [file 41598_2018_22464_MOESM1_ESM.pdf]

Identification of microRNAs involved in lipid biosynthesis and seed size in developing sea buckthorn seeds using high-throughput sequencing

Jian Ding[1], Chengjiang Ruan[1,*], Ying Guan[2] & Priti Krishna[3]

1. Key Laboratory of Biotechnology and Bioresources Utilization, Ministry of Education, Institute of Plant Resources, Dalian Minzu University, Dalian 116600, China;

2. Institute of Berries, Heilongjiang Academy of Agricultural Sciences, Suiling 152200, China;

3. School of Science and Health, Western Sydney University, Penrith NSW 2751, Australia.

*Correspondence and requests for material should be addressed to C. R. (ruan@dlnu.edu.cn)

Supplementary legends

Figure S1. Quality evaluation of the sRNA sequencing data by the FastQC software.

Table S1. Statistics of sequencing and mapping of reads on unigenes. G, G/Y, Y/O and O/R indicate the developmental stage of the fruit as indicated by color green, green/yellow, yellow/orange and orange/red, respectively; Q20 and Q30 indicate the percentage of bases with Phred values > 20 and > 30, respectively; GC indicates the GC ratio of total base number.

Table S2. Functional classifications of unigenes in developing seeds of sea buckthorn based on GO term, COG category and KEGG pathway.

Table S3. Unigenes involved in lipid metabolism based on KEGG pathway analysis.

Table S4. The information of known miRNAs in sea buckthorn seeds.

Table S5. List of known miRNAs identified in developing sea buckthorn seeds and their read counts in eight sRNA libraries. SJ1-G, SJ1-G/Y, SJ1-Y/O and SJ1-O/R indicate the developmental stage of the line 'SJ1' fruit as indicated by color green, green/yellow, yellow/orange and orange/red, respectively; XE3-G, XE3-G/Y, XE3-Y/O and XE3-O/R indicate the developmental stage of the line 'XE3' fruit as indicated by color green, green/yellow, yellow/orange and orange/red, respectively.

Table S6. The information of novel miRNAs in sea buckthorn seeds.

Table S7. List of novel miRNAs identified in developing sea buckthorn seeds and their read counts in eight sRNA libraries. SJ1-G, SJ1-G/Y, SJ1-Y/O and SJ1-O/R indicate the developmental stage of the line 'SJ1' fruit as indicated by color green, green/yellow, yellow/orange and orange/red, respectively; XE3-G, XE3-G/Y, XE3-Y/O and XE3-O/R indicate the developmental stage of the line 'XE3' fruit as indicated by color green, green/yellow, yellow/orange and orange/red, respectively.

Table S8. Differential expression miRNAs for each of 16 pairwise comparison groups. SJ1-G, SJ1-G/Y, SJ1-Y/O and SJ1-O/R indicate the developmental stage of the line 'SJ1' fruit as indicated by color green, green/yellow, yellow/orange and orange/red, respectively; XE3-G, XE3-G/Y, XE3-Y/O and XE3-O/R indicate the developmental stage of the line 'XE3' fruit as indicated by color green, green/yellow, yellow/orange and orange/red, respectively. TPM indicate the read counts of identified miRNAs were normalized as transcripts per million.

Table S9. Predicted targets for known and novel miRNAs and their functional annotations.

Table S10. Go analysis of targets of identified miRNAs in sea buckthorn seeds.

Table S11. Primers used for qRT-PCR of selected mature miRNAs.

Table S12. Primers used for qRT-PCR of predicted miRNAs target.

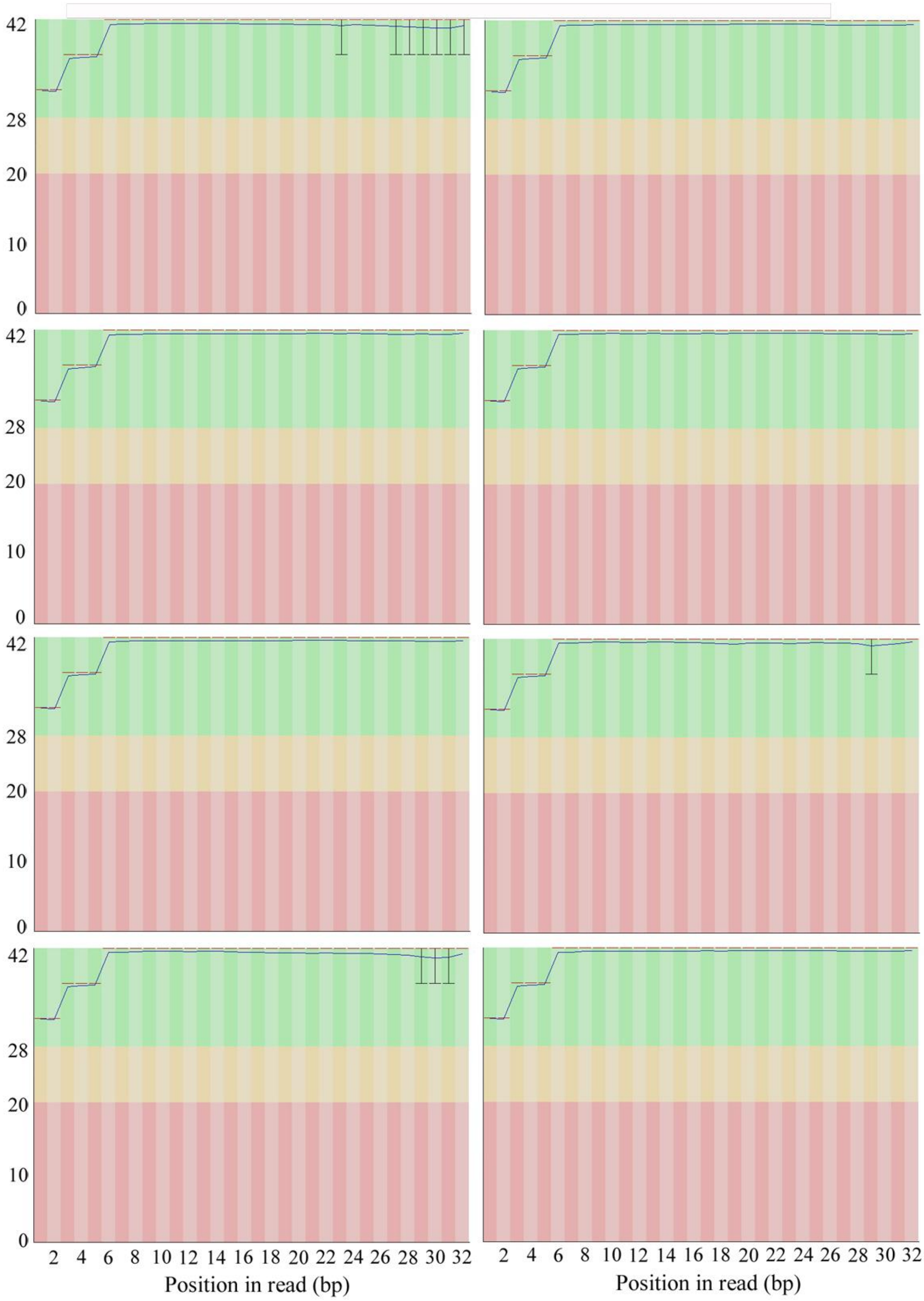

Figure S1. Quality evaluation of the sRNA sequencing data by the FastQC software.
